# Marijuana legalization and historical trends in marijuana use among US residents aged 12–25: results from the 1979–2016 National Survey on drug use and health

**DOI:** 10.1186/s12889-020-8253-4

**Published:** 2020-02-04

**Authors:** Bin Yu, Xinguang Chen, Xiangfan Chen, Hong Yan

**Affiliations:** 1 Department of Epidemiology, University of Florida, Gainesville, FL 32608 USA; 2 Department of Epidemiology and Health Statistics School of Health Sciences, Wuhan University, Wuhan, 430071 China

**Keywords:** Marijuana, Adolescents and young adults, HAPC model, United States

## Abstract

**Background:**

Marijuana is the most commonly used illicit drug in the United States. More and more states legalized medical and recreational marijuana use. Adolescents and emerging adults are at high risk for marijuana use. This ecological study aims to examine historical trends in marijuana use among youth along with marijuana legalization.

**Method:**

Data (*n* = 749,152) were from the 31-wave National Survey on Drug Use and Health (NSDUH), 1979–2016. Current marijuana use, if use marijuana in the past 30 days, was used as outcome variable. Age was measured as the chronological age self-reported by the participants, period was the year when the survey was conducted, and cohort was estimated as period subtracted age. Rate of current marijuana use was decomposed into independent age, period and cohort effects using the hierarchical age-period-cohort (HAPC) model.

**Results:**

After controlling for age, cohort and other covariates, the estimated period effect indicated declines in marijuana use in 1979–1992 and 2001–2006, and increases in 1992–2001 and 2006–2016. The period effect was positively and significantly associated with the proportion of people covered by Medical Marijuana Laws (MML) (correlation coefficients: 0.89 for total sample, 0.81 for males and 0.93 for females, all three *p* values < 0.01), but was not significantly associated with the Recreational Marijuana Laws (RML). The estimated cohort effect showed a historical decline in marijuana use in those who were born in 1954–1972, a sudden increase in 1972–1984, followed by a decline in 1984–2003.

**Conclusion:**

The model derived trends in marijuana use were coincident with the laws and regulations on marijuana and other drugs in the United States since the 1950s. With more states legalizing marijuana use in the United States, emphasizing responsible use would be essential to protect youth from using marijuana.

## Introduction

### Marijuana use and laws in the United States

Marijuana is one of the most commonly used drugs in the United States (US) [1]. In 2015, 8.3% of the US population aged 12 years and older used marijuana in the past month; 16.4% of adolescents aged 12–17 years used in lifetime and 7.0% used in the past month [2]. The effects of marijuana on a person’s health are mixed. Despite potential benefits (e.g., relieve pain) [3], using marijuana is associated with a number of adverse effects, particularly among adolescents. Typical adverse effects include impaired short-term memory, cognitive impairment, diminished life satisfaction, and increased risk of using other substances [4].

Since 1937 when the Marijuana Tax Act was issued, a series of federal laws have been subsequently enacted to regulate marijuana use, including the Boggs Act (1952), Narcotics Control Act (1956), Controlled Substance Act (1970), and Anti-Drug Abuse Act (1986) [5, 6]. These laws regulated the sale, possession, use, and cultivation of marijuana [6]. For example, the Boggs Act increased the punishment of marijuana possession, and the Controlled Substance Act categorized the marijuana into the Schedule I Drugs which have a high potential for abuse, no medical use, and not safe to use without medical supervision [5, 6]. These federal laws may have contributed to changes in the historical trend of marijuana use among youth.

### Movements to decriminalize and legalize marijuana use

Starting in the late 1960s, marijuana decriminalization became a movement, advocating reformation of federal laws regulating marijuana [7]. As a result, 11 US states had taken measures to decriminalize marijuana use by reducing the penalty of possession of small amount of marijuana [7].

The legalization of marijuana started in 1993 when Surgeon General Elder proposed to study marijuana legalization [8]. California was the first state that passed Medical Marijuana Laws (MML) in 1996 [9]. After California, more and more states established laws permitting marijuana use for medical and/or recreational purposes. To date, 33 states and the District of Columbia have established MML, including 11 states with recreational marijuana laws (RML) [9]. Compared with the legalization of marijuana use in the European countries which were more divided that many of them have medical marijuana registered as a treatment option with few having legalized recreational use [10–13], the legalization of marijuana in the US were more mixed with 11 states legalized medical and recreational use consecutively, such as California, Nevada, Washington, etc. These state laws may alter people’s attitudes and behaviors, finally may lead to the increased risk of marijuana use, particularly among young people [13]. Reported studies indicate that state marijuana laws were associated with increases in acceptance of and accessibility to marijuana, declines in perceived harm, and formation of new norms supporting marijuana use [14].

### Marijuana harm to adolescents and young adults

Adolescents and young adults constitute a large proportion of the US population. Data from the US Census Bureau indicate that approximately 60 million of the US population are in the 12–25 years age range [15]. These people are vulnerable to drugs, including marijuana [16]. Marijuana is more prevalent among people in this age range than in other ages [17]. One well-known factor for explaining the marijuana use among people in this age range is the theory of imbalanced cognitive and physical development [4]. The delayed brain development of youth reduces their capability to cognitively process social, emotional and incentive events against risk behaviors, such as marijuana use [18]. Understanding the impact of marijuana laws on marijuana use among this population with a historical perspective is of great legal, social and public health significance.

### Inconsistent results regarding the impact of marijuana laws on marijuana use

A number of studies have examined the impact of marijuana laws on marijuana use across the world, but reported inconsistent results [13]. Some studies reported no association between marijuana laws and marijuana use [14, 19–25], some reported a protective effect of the laws against marijuana use [24, 26], some reported mixed effects [27, 28], while some others reported a risk effect that marijuana laws increased marijuana use [29, 30]. Despite much information, our review of these reported studies revealed several limitations. First of all, these studies often targeted a short time span, ignoring the long period trend before marijuana legalization. Despite the fact that marijuana laws enact in a specific year, the process of legalization often lasts for several years. Individuals may have already changed their attitudes and behaviors before the year when the law is enacted. Therefore, it may not be valid when comparing marijuana use before and after the year at a single time point when the law is enacted and ignoring the secular historical trend [19, 30, 31]. Second, many studies adapted the difference-in-difference analytical approach designated for analyzing randomized controlled trials. No US state is randomized to legalize the marijuana laws, and no state can be established as controls. Thus, the impact of laws cannot be efficiently detected using this approach. Third, since marijuana legalization is a public process, and the information of marijuana legalization in one state can be easily spread to states without the marijuana laws. The information diffusion cannot be ruled out, reducing the validity of the non-marijuana law states as the controls to compare the between-state differences [31].

Alternatively, evidence derived based on a historical perspective may provide new information regarding the impact of laws and regulations on marijuana use, including state marijuana laws in the past two decades. Marijuana users may stop using to comply with the laws/regulations, while non-marijuana users may start to use if marijuana is legal. Data from several studies with national data since 1996 demonstrate that attitudes, beliefs, perceptions, and use of marijuana among people in the US were associated with state marijuana laws [29, 32].

### Age-period-cohort modeling: looking into the past with recent data

To investigate historical trends over a long period, including the time period with no data, we can use the classic age-period-cohort modeling (APC) approach. The APC model can successfully discompose the rate or prevalence of marijuana use into independent age, period and cohort effects [33, 34]. Age effect refers to the risk associated with the aging process, including the biological and social accumulation process. Period effect is risk associated with the external environmental events in specific years that exert effect on all age groups, representing the unbiased historical trend of marijuana use which controlling for the influences from age and birth cohort. Cohort effect refers to the risk associated with the specific year of birth. A typical example is that people born in 2011 in Fukushima, Japan may have greater risk of cancer due to the nuclear disaster [35], so a person aged 80 in 2091 contains the information of cancer risk in 2011 when he/she was born. Similarly, a participant aged 25 in 1979 contains information on the risk of marijuana use 25 years ago in 1954 when that person was born. With this method, we can describe historical trends of marijuana use using information stored by participants in older ages [33]. The estimated period and cohort effects can be used to present the unbiased historical trend of specific topics, including marijuana use [34, 36–38]. Furthermore, the newly established hierarchical APC (HAPC) modeling is capable of analyzing individual-level data to provide more precise measures of historical trends [33]. The HAPC model has been used in various fields, including social and behavioral science, and public health [39, 40].

Several studies have investigated marijuana use with APC modeling method [17, 41, 42]. However, these studies covered only a small portion of the decades with state marijuana legalization [17, 42]. For example, the study conducted by Miech and colleagues only covered periods from 1985 to 2009 [17]. Among these studies, one focused on a longer state marijuana legalization period, but did not provide detailed information regarding the impact of marijuana laws because the survey was every 5 years and researchers used a large 5-year age group which leads to a wide 10-year birth cohort. The averaging of the cohort effects in 10 years could reduce the capability of detecting sensitive changes of marijuana use corresponding to the historical events [41].

### Purpose of the study

In this study, we examined the historical trends in marijuana use among youth using HAPC modeling to obtain the period and cohort effects. These two effects provide unbiased and independent information to characterize historical trends in marijuana use after controlling for age and other covariates. We conceptually linked the model-derived time trends to both federal and state laws/regulations regarding marijuana and other drug use in 1954–2016. The ultimate goal is to provide evidence informing federal and state legislation and public health decision-making to promote responsible marijuana use and to protect young people from marijuana use-related adverse consequences.

## Materials and methods

### Data sources and study population

Data were derived from 31 waves of National Survey on Drug Use and Health (NSDUH), 1979–2016. NSDUH is a multi-year cross-sectional survey program sponsored by the Substance Abuse and Mental Health Services Administration. The survey was conducted every 3 years before 1990, and annually thereafter. The aim is to provide data on the use of tobacco, alcohol, illicit drug and mental health among the US population.

Survey participants were noninstitutionalized US civilians 12 years of age and older. Participants were recruited by NSDUH using a multi-stage clustered random sampling method. Several changes were made to the NSDUH after its establishment [43]. First, the name of the survey was changed from the National Household Survey on Drug Abuse (NHSDA) to NSDUH in 2002. Second, starting in 2002, adolescent participants receive $30 as incentives to improve the response rate. Third, survey mode was changed from personal interviews with self-enumerated answer sheets (before 1999) to the computer-assisted person interviews (CAPI) and audio computer-assisted self-interviews (ACASI) (since 1999). These changes may confound the historical trends [43], thus we used two dummy variables as covariates, one for the survey mode change in 1999 and another for the survey method change in 2002 to control for potential confounding effect.

### Data acquisition

Data were downloaded from the designated website (https://nsduhweb.rti.org/respweb/homepage.cfm). A database was used to store and merge the data by year for analysis. Among all participants, data for those aged 12–25 years (*n* = 749,152) were included. We excluded participants aged 26 and older because the public data did not provide information on single or two-year age that was needed for HAPC modeling (details see statistical analysis section). We obtained approval from the Institutional Review Board at the University of Florida to conduct this study.

### Variables and measurements

Current marijuana use: the dependent variable. Participants were defined as current marijuana users if they reported marijuana use within the past 30 days. We used the *variable harmonization method* to create a comparable measure across 31-wave NSDUH data [44]. Slightly different questions were used in NSDUH. In 1979–1993, participants were asked: “When was the most recent time that you used marijuana or hash?” Starting in 1994, the question was changed to “How long has it been since you last used marijuana or hashish?” To harmonize the marijuana use variable, participants were coded as current marijuana users if their response to the question indicated the last time to use marijuana was within past 30 days.

Chronological age, time period and birth cohort were the predictors. (1) Chronological age in years was measured with participants’ age at the survey. APC modeling requires the same age measure for all participants [33]. Since no data by single-year age were available for participants older than 21, we grouped all participants into two-year age groups. A total of 7 age groups, 12–13, ..., 24–25 were used. (2) Time period was measured with the year when the survey was conducted, including 1979, 1982, 1985, 1988, 1990, 1991... 2016. (3). Birth cohort was the year of birth, and it was measured by subtracting age from the survey year.

The proportion of people covered by MML: This variable was created by dividing the population in all states with MML over the total US population. The proportion was computed by year from 1996 when California first passed the MML to 2016 when a total of 29 states legalized medical marijuana use. The estimated proportion ranged from 12% in 1996 to 61% in 2016. The proportion of people covered by RML: This variable was derived by dividing the population in all states with RML with the total US population. The estimated proportion ranged from 4% in 2012 to 21% in 2016. These two variables were used to quantitatively assess the relationships between marijuana laws and changes in the risk of marijuana use.

Covariates: Demographic variables gender (male/female) and race/ethnicity (White, Black, Hispanic and others) were used to describe the study sample.

### Statistical analysis

We estimated the prevalence of current marijuana use by year using the survey estimation method, considering the complex multi-stage cluster random sampling design and unequal probability. A prevalence rate is not a simple indicator, but consisting of the impact of chronological age, time period and birth cohort, named as age, period and cohort effects, respectively. Thus, it is biased if a prevalence rate is directly used to depict the historical trend. HAPC modeling is an epidemiological method capable of decomposing prevalence rate into mutually independent age, period and cohort effects with individual-level data, while the estimated period and cohort effects provide an unbiased measure of historical trend controlling for the effects of age and other covariates. In this study, we analyzed the data using the two-level HAPC cross-classified random-effects model (CCREM) [36]:

1 $$ Logit\ \left({M}_{ijk}\right)=\mu +{\alpha}_i\Big( age\ {group}_{i\Big)}+{\beta}_j\left({period}_j\right)+{\gamma}_k\left({cohort}_k\right)+{\beta}_m\left(c{o}_{var}\right) $$

Where *M*_*ijk*_ represents the rate of marijuana use for participants in age group *i* (12–13, 14,15...), period *j* (1979, 1982,...) and birth cohort *k* (1954–55, 1956–57...); parameter *α*_*i*_ (age effect) was modeled as the fixed effect; and parameters *β*_*j*_ (period effect) and *γ*_*k*_ (cohort effect) were modeled as random effects; and *β*_*m*_ was used to control *m* covariates, including the two dummy variables assessing changes made to the NSDUH in 1999 and 2002, respectively.

The HAPC modeling analysis was executed using the PROC GLIMMIX. Sample weights were included to obtain results representing the total US population aged 12–25. A ridge-stabilized Newton-Raphson algorithm was used for parameter estimation. Modeling analysis was conducted for the overall sample, stratified by gender. The estimated age effect *α*_*i*_, period *β*_*j*_ and cohort *γ*_*k*_ (i.e., the log-linear regression coefficients) were directly plotted to visualize the pattern of change.

To gain insight into the relationship between legal events and regulations at the national level, we listed these events/regulations along with the estimated time trends in the risk of marijuana from HAPC modeling. To provide a quantitative measure, we associated the estimated period effect with the proportions of US population living with MML and RML using Pearson correlation. All statistical analyses for this study were conducted using the software SAS, version 9.4 (SAS Institute Inc., Cary, NC).

## Results

### Sample characteristics

Data for a total of 749,152 participants (12–25 years old) from all 31-wave NSDUH covering a 38-year period were analyzed. Among the total sample (Table 1), 48.96% were male and 58.78% were White, 14.84% Black, and 18.40% Hispanic.

**Table 1:** Characteristics of the study sample, overall, by gender and by race/ethnicity, the National Survey on Drug Use and Health, 1979–2016

Period	Total	Gender		Race/Ethnicity			
		Male	Female	White	Black	Hispanic	Others
1979	4209	2063	2146	3350	490	244	125
1982	2864	1404	1460	2263	358	169	74
1985	4042	1919	2123	1790	1049	1158	45
1988	4600	2199	2401	2218	1067	1217	98
1990	4229	2028	2201	2262	862	974	131
1991	15,942	7465	8477	7335	4068	3946	593
1992	14,975	7124	7851	6668	3606	4053	648
1993	12,509	6121	6388	5516	2914	3573	506
1994	10,425	5072	5353	4950	2313	2892	270
1995	8558	4074	4484	3979	2048	2260	271
1996	8904	4017	4887	3950	2207	2438	309
1997	14,083	6627	7456	6936	2408	3949	790
1998	14,096	6658	7438	6017	3172	4056	851
1999	35,766	17,514	18,252	24,182	4705	4785	2094
2000	37,293	18,351	18,942	25,035	4794	5206	2258
2001	35,090	17,182	17,908	23,400	4557	4841	2292
2002	35,437	17,320	18,117	23,534	4698	4946	2259
2003	36,587	18,210	18,377	23,162	4934	5597	2894
2004	36,769	17,991	18,778	23,344	4843	5579	3003
2005	37,154	18,067	19,087	23,080	4938	6062	3074
2006	36,246	17,974	18,272	22,137	5060	6041	3008
2007	36,044	17,851	18,193	21,908	4719	6168	3249
2008	36,495	18,021	18,474	21,546	4998	6523	3428
2009	36,288	17,793	18,495	21,404	4959	6426	3499
2010	37,469	18,512	18,957	22,331	5003	6602	3533
2011	38,447	19,165	19,282	22,564	5306	6907	3670
2012	36,014	17,720	18,294	20,640	4901	6909	3564
2013	35,878	17,642	18,236	20,114	4999	6938	3827
2014	26,669	13,176	13,493	14,691	3533	5426	3019
2015	28,138	13,688	14,450	14,940	3834	6118	3246
2016	27,932	13,822	14,110	15,140	3809	5876	3107

### Prevalence rate of current marijuana use

**Fig. 1:**
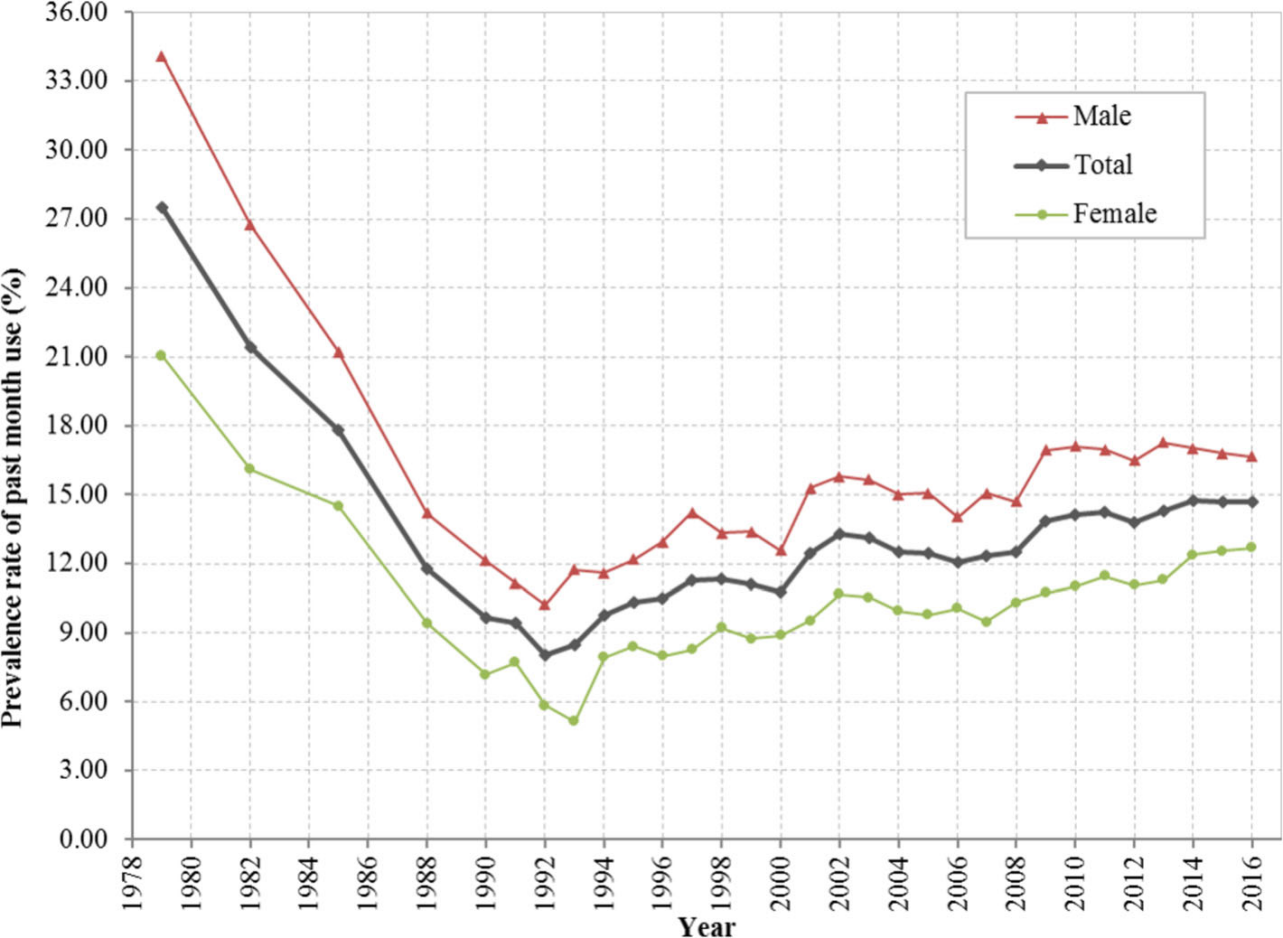
Prevalence rate (%) of current marijuana use among US residents 12 to 25 years of age during 1979–2016, overall and stratified by gender. Derived from data from the 1979–2016 National Survey on Drug Use and Health (NSDUH)

As shown in Fig. 1, the estimated prevalence rates of current marijuana use from 1979 to 2016 show a “V” shaped pattern. The rate was 27.57% in 1979, it declined to 8.02% in 1992, followed by a gradual increase to 14.70% by 2016. The pattern was the same for both male and female with males more likely to use than females during the whole period.

### HAPC modeling and results

**Fig. 2:**
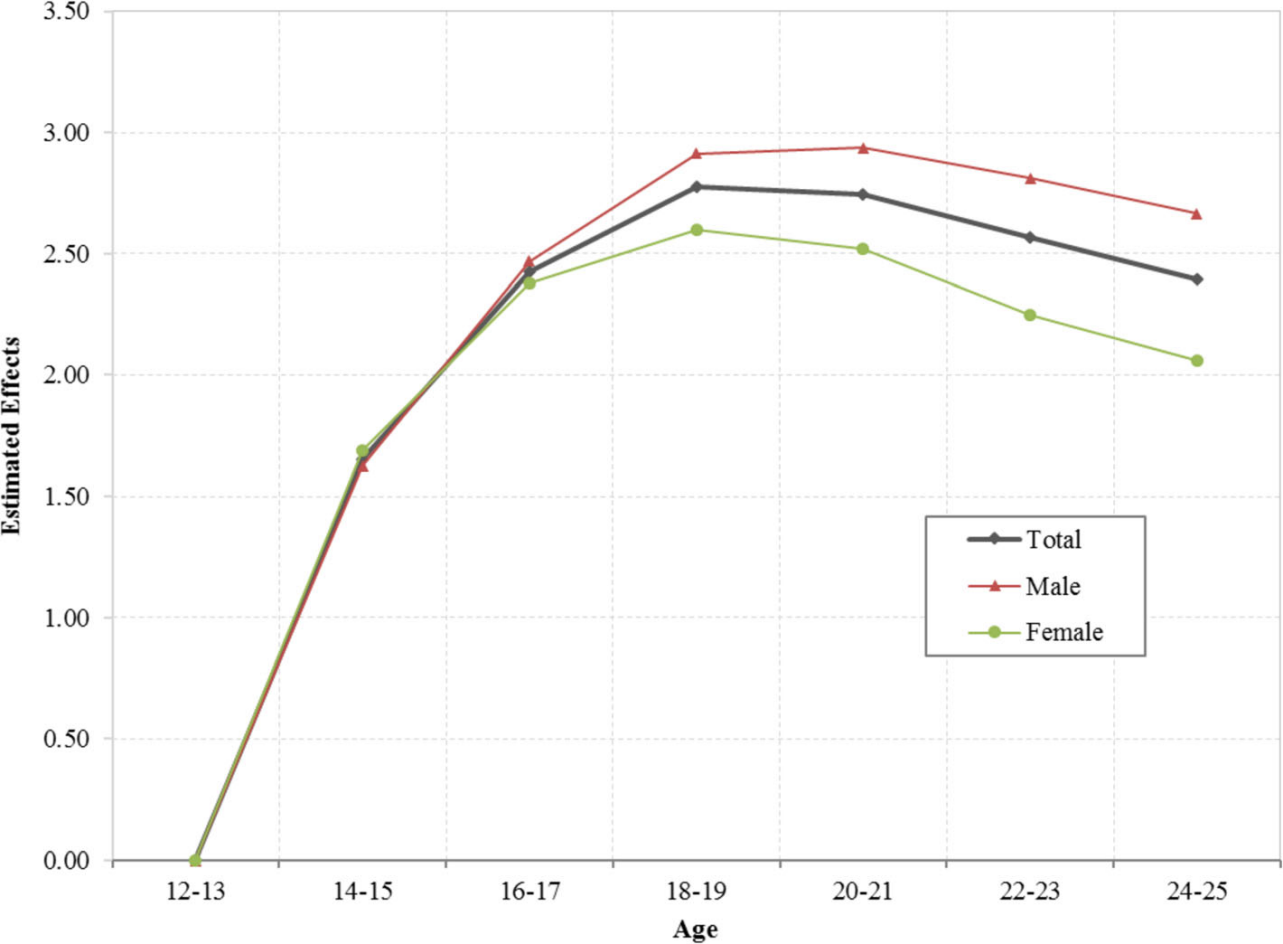
Age effect for the risk of current marijuana use, overall and stratified by male and female, estimated with hierarchical age-period-cohort modeling method with 31 waves of NSDUH data during 1979–2016. Age effect *α*_*i*_ were log-linear regression coefficients estimated using CCREM (1), see text for more details

Estimated age effects *α*_*i*_ from the CCREM [1] for current marijuana use are presented in Fig. 2. The risk by age shows a 2-phase pattern –a rapid increase phase from ages 12 to 19, followed by a gradually declining phase. The pattern was persistent for the overall sample and for both male and female subsamples.

**Fig. 3:**
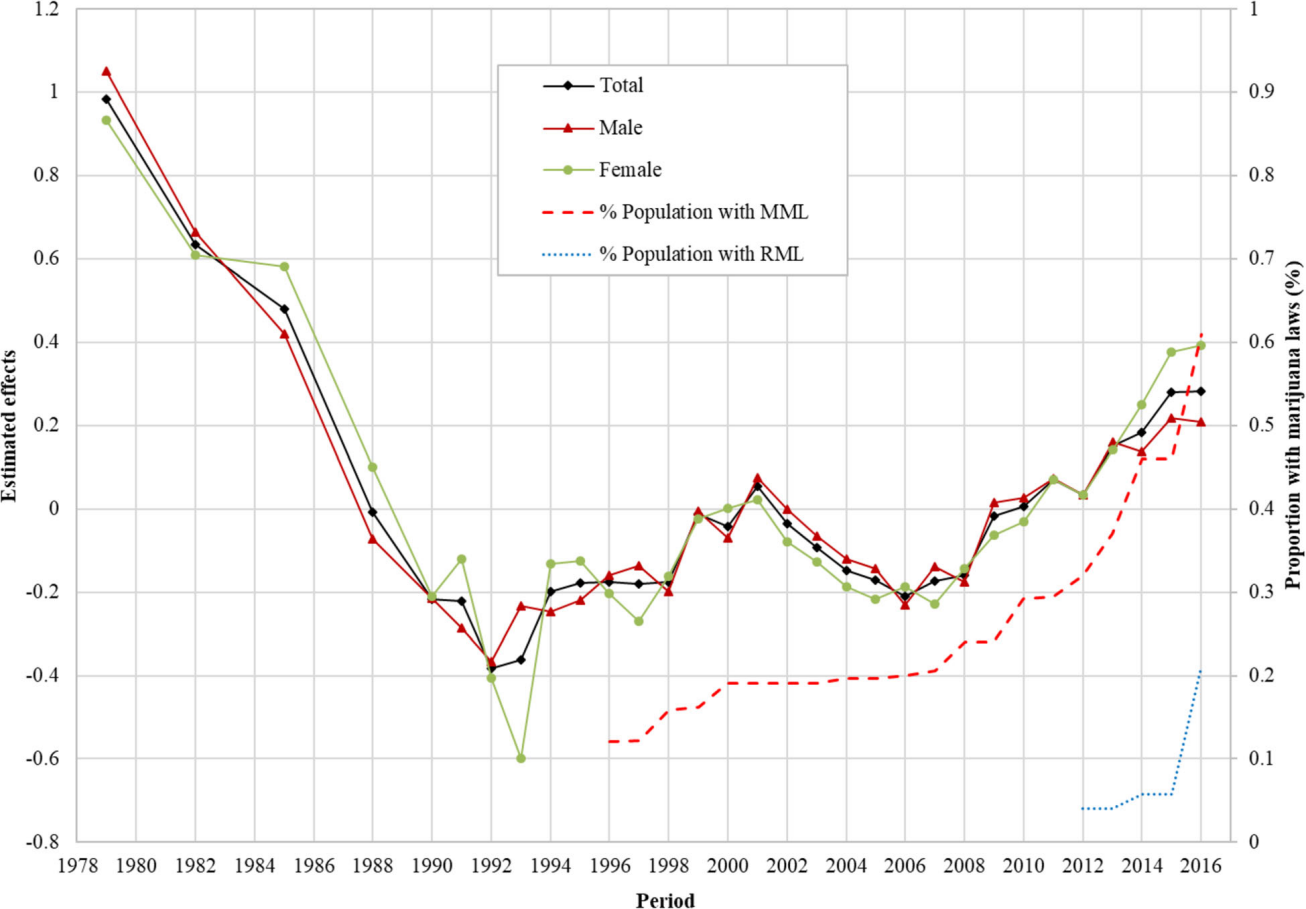
Period effect for the risk of marijuana use for US adolescents and young adults, overall and by male/female estimated with hierarchical age-period-cohort modeling method and its correlation with the proportion of US population covered by Medical Marijuana Laws and Recreational Marijuana Laws. Period effect *β*_*j*_ were log-linear regression coefficients estimated using CCREM (1), see text for more details

The estimated period effects *β*_*j*_ from the CCREM [1] are presented in Fig. 3. The period effect reflects the risk of current marijuana use due to significant events occurring over the period, particularly federal and state laws and regulations. After controlling for the impacts of age, cohort and other covariates, the estimated period effect indicates that the risk of current marijuana use had two declining trends (1979–1992 and 2001–2006), and two increasing trends (1992–2001 and 2006–2016). Epidemiologically, the time trends characterized by the estimated period effects in Fig. 3 are more valid than the prevalence rates presented in Fig. 1 because the former was adjusted for confounders while the later was not.

Correlation of the period effect with proportions of the population covered by marijuana laws: The Pearson correlation coefficient of the period effect with the proportions of US population covered by MML during 1996–2016 was 0.89 for the total sample, 0.81 for male and 0.93 for female, respectively (*p* < 0.01 for all). The correlation between period effect and proportion of US population covered by RML was 0.64 for the total sample, 0.59 for male and 0.49 for female (*p* > 0.05 for all).

**Fig. 4:**
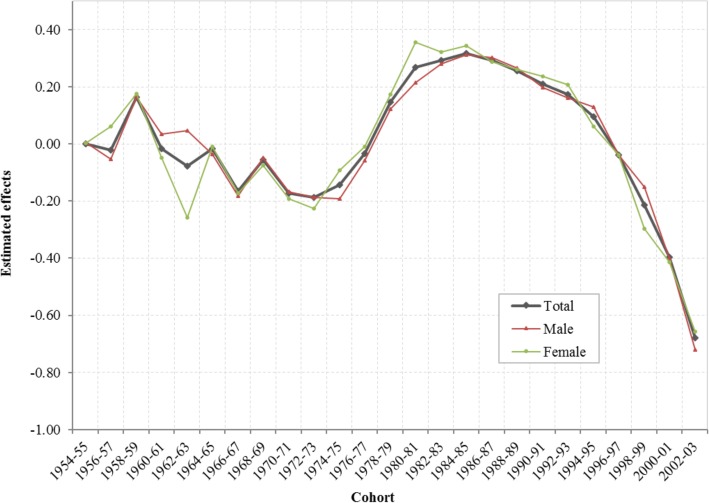
Cohort effect for the risk of marijuana use among US adolescents and young adults born during 1954–2003, overall and by male/female, estimated with hierarchical age-period-cohort modeling method. Cohort effect *γ*_*k*_ were log-linear regression coefficients estimated using CCREM (1), see text for more details

Likewise, the estimated cohort effects *γ*_*k*_ from the CCREM [1] are presented in Fig. 4. The cohort effect reflects changes in the risk of current marijuana use over the period indicated by the year of birth of the survey participants after the impacts of age, period and other covariates are adjusted. Results in the figure show three distinctive cohorts with different risk patterns of current marijuana use during 1954–2003: (1) the Historical Declining Cohort (HDC): those born in 1954–1972, and characterized by a gradual and linear declining trend with some fluctuations; (2) the Sudden Increase Cohort (SIC): those born from 1972 to 1984, characterized with a rapid almost linear increasing trend; and (3) the Contemporary Declining Cohort (CDC): those born during 1984 and 2003, and characterized with a progressive declining over time. The detailed results of HAPC modeling analysis were also shown in Additional file 1: Table S1.

## Discussion

This study provides new data regarding the risk of marijuana use in youth in the US during 1954–2016. This is a period in the US history with substantial increases and declines in drug use, including marijuana; accompanied with many ups and downs in legal actions against drug use since the 1970s and progressive marijuana legalization at the state level from the later 1990s till today (see Additional file 1: Table S2). Findings of the study indicate four-phase period effect and three-phase cohort effect, corresponding to various historical events of marijuana laws, regulations and social movements.

### Coincident relationship between the period effect and legal drug control

The period effect derived from the HAPC model provides a net effect of the impact of time on marijuana use after the impact of age and birth cohort were adjusted. Findings in this study indicate that there was a progressive decline in the period effect during 1979 and 1992. This trend was corresponding to a period with the strongest legal actions at the national level, the War on Drugs by President Nixon (1969–1974) President Reagan (1981–1989) [45], and President Bush (1989) [45],and the Anti-Drug Abuse Act (1986) [5].

The estimated period effect shows an increasing trend in 1992–2001. During this period, President Clinton advocated for the use of treatment to replace incarceration (1992) [45], Surgeon General Elders proposed to study marijuana legalization (1993–1994) [8], President Clinton’s position of the need to re-examine the entire policy against people who use drugs, and decriminalization of marijuana (2000) [45] and the passage of MML in eight US states.

The estimated period effect shows a declining trend in 2001–2006. Important laws/regulations include the Student Drug Testing Program promoted by President Bush, and the broadened the public schools’ authority to test illegal drugs among students given by the US Supreme Court (2002) [46].

The estimated period effect increases in 2006–2016. This is the period when the proportion of the population covered by MML progressively increased. This relation was further proved by a positive correlation between the estimated period effect and the proportion of the population covered by MML. In addition, several other events occurred. For example, over 500 economists wrote an open letter to President Bush, Congress and Governors of the US and called for marijuana legalization (2005) [47], and President Obama ended the federal interference with the state MML, treated marijuana as public health issues, and avoided using the term of “War on Drugs” [45]. The study also indicates that the proportion of population covered by RML was positively associated with the period effect although not significant which may be due to the limited number of data points of RML. Future studies may follow up to investigate the relationship between RML and rate of marijuana use.

### Coincident relationship between the cohort effect and legal drug control

Cohort effect is the risk of marijuana use associated with the specific year of birth. People born in different years are exposed to different laws, regulations in the past, therefore, the risk of marijuana use for people may differ when they enter adolescence and adulthood. Findings in this study indicate three distinctive cohorts: HDC (1954–1972), SIC (1972–1984) and CDC (1984–2003). During HDC, the overall level of marijuana use was declining. Various laws/regulations of drug use in general and marijuana in particular may explain the declining trend. First, multiple laws passed to regulate the marijuana and other substance use before and during this period remained in effect, for example, the Marijuana Tax Act (1937), the Boggs Act (1952), the Narcotics Control Act (1956) and the Controlled Substance Act (1970). Secondly, the formation of government departments focusing on drug use prevention and control may contribute to the cohort effect, such as the Bureau of Narcotics and Dangerous Drugs (1968) [48]. People born during this period may be exposed to the macro environment with laws and regulations against marijuana, thus, they may be less likely to use marijuana.

Compared to people born before 1972, the cohort effect for participants born during 1972 and 1984 was in coincidence with the increased risk of using marijuana shown as SIC. This trend was accompanied by the state and federal movements for marijuana use, which may alter the social environment and public attitudes and beliefs from prohibitive to acceptive. For example, seven states passed laws to decriminalize the marijuana use and reduced the penalty for personal possession of small amount of marijuana in 1976 [7]. Four more states joined the movement in two subsequent years [7]. People born during this period may have experienced tolerated environment of marijuana, and they may become more acceptable of marijuana use, increasing their likelihood of using marijuana.

A declining cohort CDC appeared immediately after 1984 and extended to 2003. This declining cohort effect was corresponding to a number of laws, regulations and movements prohibiting drug use. Typical examples included the War on Drugs initiated by President Nixon (1980s), the expansion of the drug war by President Reagan (1980s), the highly-publicized anti-drug campaign “Just Say No” by First Lady Nancy Reagan (early 1980s) [45], and the Zero Tolerance Policies in mid-to-late 1980s [45], the Anti-Drug Abuse Act (1986) [5], the nationally televised speech of War on Drugs declared by President Bush in 1989 and the escalated War on Drugs by President Clinton (1993–2001) [45]. Meanwhile many activities of the federal government and social groups may also influence the social environment of using marijuana. For example, the Federal government opposed to legalize the cultivation of industrial hemp, and Federal agents shut down marijuana sales club in San Francisco in 1998 [48]. Individuals born in these years grew up in an environment against marijuana use which may decrease their likelihood of using marijuana when they enter adolescence and young adulthood.

## Conclusion

This study applied the age-period-cohort model to investigate the independent age, period and cohort effects, and indicated that the model derived trends in marijuana use among adolescents and young adults were coincident with the laws and regulations on marijuana use in the United States since the 1950s. With more states legalizing marijuana use in the United States, emphasizing responsible use would be essential to protect youth from using marijuana.

### Limitations

This study has limitations. First, study data were collected through a household survey, which is subject to underreporting. Second, no causal relationship can be warranted using cross-sectional data, and further studies are needed to verify the association between the specific laws/regulation and the risk of marijuana use. Third, data were available to measure single-year age up to age 21 and two-year age group up to 25, preventing researchers from examining the risk of marijuana use for participants in other ages. Lastly, data derived from NSDUH were nation-wide, and future studies are needed to analyze state-level data and investigate the between-state differences. Although a systematic review of all laws and regulations related to marijuana and other drugs is beyond the scope of this study, findings from our study provide new data from a historical perspective much needed for the current trend in marijuana legalization across the nation to get the benefit from marijuana while to protect vulnerable children and youth in the US. It provides an opportunity for stack-holders to make public decisions by reviewing the findings of this analysis together with the laws and regulations at the federal and state levels over a long period since the 1950s.

## Supplementary information

**Additional file 1: Table S1.** Estimated Age, Period, Cohort Effects for the Trend of Marijuana Use in Past Month among Adolescents and Emerging Adults Aged 12 to 25 Years, NSDUH, 1979-2016. **Table S2.** Laws at the federal and state levels related to marijuana use.

## Data Availability

The data of the study are available from the designated repository (https://nsduhweb.rti.org/respweb/homepage.cfm).

## Abbreviations

ACASI: Audio computer-assisted self-interviews
APC: Age-period-cohort modeling
CAPI: Computer-assisted person interviews
CCREM: Cross-classified random-effects model
CDC: Contemporary Declining Cohort
HAPC: Hierarchical age-period-cohort
HDC: Historical Declining Cohort
MML: Medical Marijuana Laws
NHSDA: National Household Survey on Drug Abuse
NSDUH: National Survey on Drug Use and Health
RML: Recreational Marijuana Laws
SIC: Sudden Increase Cohort
US: The United States
