# Supplementary material for: Marijuana legalization and historical trends in marijuana use among US residents aged 12–25: results from the 1979–2016 National Survey on drug use and health
Source: BMC Public Health. 2020 Feb 4;20:156. doi: 10.1186/s12889-020-8253-4 (PMC6998313; doi:10.1186/s12889-020-8253-4)
Supplement: Supplementary file 1 — Additional file 1: Table S1. Estimated Age, Period, Cohort Effects for the Trend of Marijuana Use in Past Month among Adolescents and Emerging Adults Aged 12 to 25 Years, NSDUH, 1979-2016. Table S2. Laws at the federal and state levels related to marijuana use. [file 12889_2020_8253_MOESM1_ESM.docx]

**Table S1. Estimated Age, Period, Cohort Effects for the Trend of Marijuana Use in Past Month among Adolescents and Emerging Adults Aged 12 to 25 Years, NSDUH, 1979-2016**

|  | Total | |  | Male | |  | Female | |
| --- | --- | --- | --- | --- | --- | --- | --- | --- |
|  | Effects | 95% CI |  | Effects | 95% CI |  | Effects | 95% CI |
| **Age** |  |  |  |  |  |  |  |  |
| 12 | 0.00 | N/A |  | 0.00 | N/A |  | 0.00 | N/A |
| 14 | 1.65 | 1.64, 1.67 |  | 1.63 | 1.61, 1.64 |  | 1.69 | 1.68, 1.70 |
| 16 | 2.43 | 2.40, 2.45 |  | 2.47 | 2.44, 2.49 |  | 2.38 | 2.35, 2.41 |
| 18 | 2.77 | 2.74, 2.81 |  | 2.91 | 2.87, 2.95 |  | 2.60 | 2.56, 2.64 |
| 20 | 2.75 | 2.70, 2.80 |  | 2.93 | 2.89, 2.99 |  | 2.52 | 2.47, 2.58 |
| 22 | 2.56 | 2.50, 2.63 |  | 2.81 | 2.75, 2.88 |  | 2.25 | 2.18, 2.32 |
| 24 | 2.40 | 2.32, 2.47 |  | 2.67 | 2.59, 2.74 |  | 2.06 | 1.98, 2.14 |
| **Period** |  |  |  |  |  |  |  |  |
| 1979 | 0.98 | 0.81, 1.16 |  | 1.05 | 0.87, 1.23 |  | 0.93 | 0.74, 1.13 |
| 1982 | 0.63 | 0.46, 0.81 |  | 0.66 | 0.49, 0.84 |  | 0.61 | 0.42, 0.80 |
| 1985 | 0.48 | 0.31, 0.65 |  | 0.42 | 0.25, 0.59 |  | 0.58 | 0.40, 0.76 |
| 1988 | -0.01 | -0.17, 0.16 |  | -0.07 | -0.24, 0.09 |  | 0.10 | -0.08, 0.28 |
| 1990 | -0.22 | -0.38, -0.05 |  | -0.21 | -0.38, -0.05 |  | -0.21 | -0.39, -0.03 |
| 1991 | -0.22 | -0.38, -0.06 |  | -0.29 | -0.45, -0.12 |  | -0.12 | -0.30, 0.06 |
| 1992 | -0.38 | -0.55, -0.22 |  | -0.37 | -0.53, -0.20 |  | -0.40 | -0.58, -0.23 |
| 1993 | -0.36 | -0.53, -0.20 |  | -0.23 | -0.40, -0.07 |  | -0.60 | -0.78, -0.42 |
| 1994 | -0.20 | -0.36, -0.03 |  | -0.25 | -0.41, -0.08 |  | -0.13 | -0.31, 0.05 |
| 1995 | -0.18 | -0.34, -0.01 |  | -0.22 | -0.39, -0.05 |  | -0.13 | -0.31, 0.05 |
| 1996 | -0.17 | -0.34, -0.01 |  | -0.16 | -0.33, 0.01 |  | -0.20 | -0.38, -0.02 |
| 1997 | -0.18 | -0.35, -0.01 |  | -0.14 | -0.31, 0.03 |  | -0.27 | -0.45, -0.09 |
| 1998 | -0.18 | -0.35, -0.01 |  | -0.20 | -0.37, -0.03 |  | -0.16 | -0.34, 0.02 |
| 1999 | -0.01 | -0.55, 0.33 |  | -0.01 | -0.35, 0.34 |  | -0.02 | -0.39, 0.34 |
| 2000 | -0.04 | -0.38, 0.30 |  | -0.07 | -0.41, 0.27 |  | 0.01 | -0.37, 0.37 |
| 2001 | 0.05 | -0.29, 0.39 |  | 0.08 | -0.27, 0.42 |  | 0.02 | -0.35, 0.39 |
| 2002 | -0.03 | -0.19, 0.12 |  | -0.001 | -0.16, 0.16 |  | -0.08 | -0.25, 0.09 |
| 2003 | -0.09 | -0.25, 0.06 |  | -0.07 | -0.22, 0.09 |  | -0.13 | -0.30, 0.04 |
| 2004 | -0.15 | -0.30, 0.01 |  | -0.12 | -0.28, 0.04 |  | -0.19 | -0.36, -0.02 |
| 2005 | -0.17 | -0.33, -0.02 |  | -0.14 | -0.30, 0.01 |  | -0.22 | -0.38, -0.05 |
| 2006 | -0.21 | -0.36, -0.06 |  | -0.23 | -0.39, -0.08 |  | -0.19 | -0.35, -0.02 |
| 2007 | -0.17 | -0.33, -0.02 |  | -0.14 | -0.29, 0.01 |  | -0.23 | -0.38, -0.06 |
| 2008 | -0.16 | -0.31, -0.01 |  | -0.18 | -0.33, -0.02 |  | -0.14 | -0.31, 0.02 |
| 2009 | -0.02 | -0.17, 0.14 |  | 0.02 | -0.14, 0.17 |  | -0.06 | -0.23, 0.10 |
| 2010 | 0.01 | -0.15, 0.16 |  | 0.03 | -0.13, 0.18 |  | -0.03 | -0.19, 0.13 |
| 2011 | 0.07 | -0.08, 0.22 |  | 0.07 | -0.08, 0.23 |  | 0.07 | -0.10, 0.24 |
| 2012 | 0.03 | -0.12, 0.19 |  | 0.03 | -0.12, 0.19 |  | 0.03 | -0.13, 0.20 |
| 2013 | 0.15 | -0.01, 0.31 |  | 0.16 | 0.01, 0.32 |  | 0.14 | -0.03, 0.31 |
| 2014 | 0.18 | 0.03, 0.34 |  | 0.14 | -0.02, 0.29 |  | 0.25 | 0.08, 0.42 |
| 2015 | 0.28 | 0.12, 0.44 |  | 0.22 | 0.06, 0.38 |  | 0.38 | 0.21, 0.55 |
| 2016 | 0.28 | 0.12, 0.44 |  | 0.21 | 0.05, 0.37 |  | 0.39 | 0.22, 0.56 |
| **Cohort** |  |  |  |  |  |  |  |  |
| 1954 | 0.00 | -0.18, 0.18 |  | 0.01 | -0.17, 0.18 |  | 0.01 | -0.19, 0.20 |
| 1956 | -0.02 | -0.19, 0.15 |  | -0.05 | -0.22, 0.12 |  | 0.06 | -0.12, 0.24 |
| 1958 | 0.16 | 0.01, 0.32 |  | 0.16 | 0.01, 0.32 |  | 0.18 | 0.01, 0.34 |
| 1960 | -0.02 | -0.16, 0.13 |  | 0.04 | -0.11, 0.18 |  | -0.05 | -0.21, 0.11 |
| 1962 | -0.08 | -0.21, 0.06 |  | 0.05 | -0.09, 0.18 |  | -0.26 | -0.41, -0.11 |
| 1964 | -0.02 | -0.15, 0.12 |  | -0.04 | -0.17, 0.09 |  | -0.01 | -0.15, 0.13 |
| 1966 | -0.16 | -0.28, -0.04 |  | -0.18 | -0.30, -0.06 |  | -0.17 | -0.30, -0.04 |
| 1968 | -0.06 | -0.17, 0.06 |  | -0.05 | -0.16, 0.07 |  | -0.08 | -0.20, 0.05 |
| 1970 | -0.17 | -0.28, -0.06 |  | -0.17 | -0.27, -0.06 |  | -0.19 | -0.31, -0.08 |
| 1972 | -0.19 | -0.29, -0.09 |  | -0.19 | -0.29, -0.08 |  | -0.23 | -0.34, -0.12 |
| 1974 | -0.14 | -0.24, -0.05 |  | -0.19 | -0.29, -0.09 |  | -0.09 | -0.20, 0.01 |
| 1976 | -0.03 | -0.13, 0.06 |  | -0.06 | -0.15, 0.04 |  | -0.01 | -0.11, 0.09 |
| 1978 | 0.15 | 0.05, 0.24 |  | 0.12 | 0.03, 0.22 |  | 0.17 | 0.07, 0.28 |
| 1980 | 0.27 | 0.17, 0.36 |  | 0.21 | 0.12, 0.31 |  | 0.36 | 0.25, 0.46 |
| 1982 | 0.29 | 0.20, 0.39 |  | 0.28 | 0.18, 0.38 |  | 0.32 | 0.22, 0.43 |
| 1984 | 0.32 | 0.22, 0.42 |  | 0.31 | 0.21, 0.42 |  | 0.34 | 0.23, 0.45 |
| 1986 | 0.29 | 0.19, 0.40 |  | 0.30 | 0.19, 0.41 |  | 0.29 | 0.17, 0.40 |
| 1988 | 0.26 | 0.14, 0.37 |  | 0.27 | 0.15, 0.38 |  | 0.26 | 0.14, 0.38 |
| 1990 | 0.21 | 0.09, 0.33 |  | 0.20 | 0.08, 0.32 |  | 0.24 | 0.11, 0.37 |
| 1992 | 0.17 | 0.05, 0.30 |  | 0.16 | 0.03, 0.29 |  | 0.21 | 0.07, 0.35 |
| 1994 | 0.10 | -0.04, 0.23 |  | 0.13 | -0.01, 0.27 |  | 0.06 | -0.09, 0.21 |
| 1996 | -0.04 | -0.18, 0.11 |  | -0.04 | -0.19, 0.11 |  | -0.04 | -0.20, 0.12 |
| 1998 | -0.21 | -0.37, -0.06 |  | -0.15 | -0.31, 0.01 |  | -0.30 | -0.47, -0.13 |
| 2000 | -0.40 | -0.56, -0.23 |  | -0.40 | -0.57, -0.23 |  | -0.41 | -0.60, -0.23 |
| 2002 | -0.68 | -0.86, -0.50 |  | -0.72 | -0.90, -0.54 |  | -0.66 | -0.85, -0.46 |

**Table S2. Laws at the federal and state levels related to marijuana use.**

| **Year** | **Laws/regulations related to marijuana use at federal and state level** |
| --- | --- |
| 1952 | Boggs Act |
| 1956 | Narcotics Control Act |
| 1970 | Controlled Substance Act |
| 1976 | California, Colorado, Maine, Minnesota, Missouri, Ohio, Oregon decriminalize marijuana use |
| 1978 | North Carolina, New York decriminalize marijuana use |
| 1979 | Nebraska decriminalize marijuana use |
| 1986 | Anti-Drug Abuse Act |
| 1996 | California passed Medical Marijuana Laws |
| 1998 | Alaska, Oregon, and Washington passed Medical Marijuana Laws |
| 1999 | Maine passed Medical Marijuana Laws |
| 2000 | Colorado, Hawaii, Nevada passed Medical Marijuana Laws |
| 2004 | Montana and Vermont passed Medical Marijuana Laws |
| 2006 | Rhode Island passed Medical Marijuana Laws |
| 2007 | New Mexico passed Medical Marijuana Laws |
| 2008 | Michigan passed Medical Marijuana Laws |
| 2010 | Arizona, New Jersey, and Washington DC passed Medical Marijuana Laws |
| 2011 | Delaware passed Medical Marijuana Laws |
| 2012 | Connecticut, Massachusetts passed Medical Marijuana Laws, Colorado and Washington passed Recreational Marijuana Laws |
| 2013 | Illinois, New Hampshire passed Medical Marijuana Laws |
| 2014 | Maryland, Minnesota passed MML, Alaska, Oregon and DC passed Recreational Marijuana Laws |
| 2016 | Arkansas, Florida, Louisiana, North Dakota, Ohio, Pennsylvania passed Medical Marijuana Laws, California, Nevada, Maine and Massachusetts passed Recreational Marijuana Laws |
| 2017 | West Virginia passed Medical Marijuana Laws |
| 2018 | Oklahoma, Missouri and Utah passed the State Medical Marijuana Laws, Vermont passed the State Recreational Marijuana Laws |

References:

<https://healthcare.findlaw.com/patient-rights/medical-marijuana-laws-by-state.html>

<https://medicalmarijuana.procon.org/view.resource.php?resourceID=000881#Florida>

<https://www.marijuanadoctors.com/medical-marijuana/la/qualification/>
